# Bibliometric analysis of genomic selection in breeding of animal from 1993 to 2024: global trends and advancements

**DOI:** 10.3389/fgene.2024.1402140

**Published:** 2024-10-24

**Authors:** Şenol Çelik

**Affiliations:** Biometry Genetics Unit, Department of Animal Science, Agricultural Faculty, Bingöl University, Bingöl, Türkiye

**Keywords:** bibliometric analysis, research on evaluation, literature review, genomic selection, trend

## Abstract

Animal breeding became a difficult science when numerous genes influenced economically significant features. The major source of genetic improvement is selection, and as such, the large generation intervals in these strategies lead to reduced rates of improvement. Therefore, breeding control, genetic improvement research, and selection processes are accelerated by genomic selection. This article regarding global research interest trends in genomic selection in animal breeding themes was examined using bibliometric analysis, which employed papers from 1993 to 2024 from the SCI-Expanded, SSCI, AHCI, and E-SCI indexes. Over the period of 31 years, the first 3,181 published articles on genomic selection in animal breeding were gathered. Additionally, the study displays trends in co-authorships according to nations and academic institutions as well as co-occurrences of author keywords. There have been more articles since 2010 about the use of genomic selection in animal breeding, building up a sizable library of work that will last until 2024. Among the top academics in the field are Calus MPL, Li J, and Wang Y. The most productive institutions were The United Kingdom’s University of Edinburgh, Aarhus University (Denmark) and China Agricultural University. The current hotspots in this field of study include “selection,” and “association,” according to keyword co-occurrence and frequency analysis. China, the United States, Brazil, Canada, and United Kingdom are the top five countries that produced the most papers with the highest levels of international collaboration and networking. The main topics of current study include prediction, accuracy, association, traits, and selection. New techniques for selection, prediction, accuracy, traits, and association were developed as the discipline matured. Research collaborations across countries, institutions, and writers promote knowledge sharing, effective issue resolution, and superior outcomes.

## 1 Introduction

Genomic selection employs dense markers that cover the entire genome, addressing the majority of genetic variations across animals (Meuwissen et al., 2001). Genomic selection (GS) assumes that all markers are related to a gene that influences the characteristic and focuses on assessing their effect rather than testing its significance. Three technological breakthroughs have led to the widespread use of DNA information in animal breeding: the development of genomic selection technology, the discovery of massive numbers of genetic markers (single nucleotide polymorphisms; SNPs), and high-throughput technology to genotype animals for (hundreds of) thousands of SNPs in a cost-effective manner (Meuwissen et al., 2016).

The basic idea behind genomic selection which was initially presented by Meuwissen et al. (2001) is that breeding values can be estimated using data from a large number of markers without requiring precise knowledge of the location of particular genes on the genome. It is expected that a gene or DNA fragment of interest will always be close to a tens of thousands of SNP, carefully selected to be representative of the entire genome. The substantial linkage disequilibrium that exists between one or more SNP and a causal mutation can then be used to explain a significant portion of the variation of the observed trait. Thus, getting access to a sizable population of animals a training population or reference population that exhibits precise phenotypes for the trait(s) is the first stage in the genomic selection process (Eggen, 2012).

Genomic selection techniques were also used in a Magalhães et al. (2019) study to examine a large number of SNPs dispersed throughout the animal genome in order to predict breeding values without needing to know the precise location of genes.

While population and quantitative genetics, as well as the science of genetics, were known long before animal breeding, the mainstay of breeding programs has been the simple process of choosing and mating the best individuals based on their own or their relatives’ performance. Thanks to the availability of genetic data, a revolution known as “genomic selection” is taking place, whereby molecular information is utilized to improve responsiveness. Breeding value projections continue to use numerous loci across the genome and are, in fact, mostly consistent with additive and more precisely infinitesimal model assumptions (Hill, 2014).

The world’s population demand and animal output are significantly out of sync. Results from experiments and simulations indicate that genomic selection for young animals without individual performance can predict breeding values with high accuracy. Genomic selection is a kind of marker-assisted selection in which all quantitative trait loci are in linkage disequilibrium with at least one marker through the use of genetic markers throughout the whole genome. Early animal selection makes it possible to develop innovative breeding techniques that increase genetic advancement while cutting expenses. For cattle breeding firms, genomic selection is the way of the future; it increases genetic gain through less genetic interval and improved dependability (Ibtisham et al., 2017).

Genomic information-assisted genetic assessment has the potential to reduce the average inbreeding coefficient and increase the genetic gain rate in dairy breeding operations. Based on a discount rate of 6.32% aa, economic viability indicators revealed that only the BLUP and GBLUP/GBLUPI methods, which had zootechnical control and genetic evaluation, either conventionally or with the use of genomic information, were economically feasible (de Oliveira Seno et al., 2018). In a study, alternative genomic selection and classic BLUP breeding techniques were evaluated to enhance feed efficiency in simulated Norwegian Red dairy cattle herds. The change in genetic gain with time and feasible selection accuracy were investigated for milk yield and residual feed intake as a measure of feed efficiency. When feed efficiency was included in genomic BLUP schemes, excellent selection accuracies were achieved for genomic selection, and all genomic BLUP schemes produced more genetic gain for feed efficiency than BLUP using a pedigree relationship matrix (Wallen et al., 2017).

Despite the fact that genomic selection has been effective in raising rates of genetic gain, relatively little was still understood about the genetic structure underlying quantitative variation. The infinitesimal model for quantitative traits suggests that almost all economic features are influenced by a relatively large number of genes. The selection goals will prioritize health, reproduction, efficiency, and environmentally friendly production with lower waste and gas emissions over qualities related to milk production. New mutations, alterations in selection objectives and management, and an increase in the frequency of uncommon alleles are the factors that sustain genetic variation for economic attributes (Weller et al., 2017).

Two innovations marked the start of the genomic selection revolution. The first was the recent sequencing of the bovine genome, which produced thousands of single nucleotide polymorphisms (SNPs) representing DNA markers. The second breakthrough, known as genomic selection, showed that breeding values may be predicted from dense marker data alone, allowing for extremely accurate selection decisions (Meuwissen et al., 2001).

Genomic selection became a reality in 2008 with the release of high-density SNP chips, which included >50,000 (50 K) markers (Van Tassell et al., 2008). The United States, Canada, Great Britain, Ireland, New Zealand, Australia, France, the Netherlands, Germany, and the Scandinavian nations have all effectively adopted this technology (Silva et al., 2014).

Breeding values in genomic selection are assessed using thousands of DNA markers rather than individual performance and family history. It is possible to reduce the generation interval for dairy cattle by doing away with the progeny test by using molecular markers to provide precise breeding values for animals of both sexes early in life (Schaeffer, 2006).

A single breeding organization conducted selection in the Saanen and French Alpine dairy goat breeds, according to a study. A combination index derived from the Estimated Breeding Value (EBV) for milk yield, fat and protein yields, fat and protein contents, and different udder-type traits was used to select for these traits (Clément et al., 2006; Rupp et al., 2011).

There are studies on bibliometric analysis of genomic selection studies related to animal science. The number of authors per paper in the study by Önder and Tırınk (2022) demonstrated that genetic selection is a collaborative endeavor, with tasks best performed by a group of scientists working together. Merely one-third of the studies on genetic selection had anything to do with animal science. The explanation for this could be that breeding plants is easier than dealing with mammals, which makes generation intervals more difficult. Upon examination of the article issues, it was observed that the majority of the articles dealt with dairy science.

The thorough bibliometric analysis of 335 documents scanned in the Web of Science (WoS) database in the field of next-generation sequence applications in livestock between 2009 and 2023 was carried out by Kaplan and Altay (2023).

In genomic selection for animal breeding, traits such as conservation of genetic resources, genetic progress, molecular genetic tools, examination of genetic variation and breeding value estimation are determined. This study focus about metric. In order to determine the most affected nations, affiliations, authors, and current research directions in the field of genomic selection in animal breeding, this study conducted a bibliometrics analysis of pertinent literature from 1993 to 2024 using the Web of Science Core Collection database.

## 2 Materials and methods

### 2.1 Materials

The studies taken into consideration for this investigation were assessed using the bibliometric analysis approach. The Web of Science core collection database and publications included in the AHCI, ESCI, SSCI, and SCI-EXPANDED indexes provided the study’s data. Between 1993 and 2024, the term “genomic selection in animal breeding” was looked up in the study. Only papers on the topic under discussion were discussed in this study. Enough data was gathered to characterize the research hotspots, scientific environment, and other analyses performed in this paper. On 29 April 2024, 3,181 data points were collected by data extraction, loading, and conversion following refinement. The bibliometric analysis was performed using the Bibliometrix package in the R programming language (Aria and Cuccurullo, 2017). The distribution of the obtained documents according to research areas according to WoS was reported and the majority of the publications were in the subject area of ‘Agriculture Dairy Animal Science’ (n = 1777, 55.86%). This was followed by ‘Genetics Heredity’ (n = 1576, 49.54%), ‘Veterinary Sciences’ (n = 466, 14.65%), ‘Multidisciplinary Sciences’ (n = 313, 9.84%), ‘Biotechnology Applied Microbiology’ (n = 307, 9.65%), ‘Zoology’ (n = 214, 6.73%), ‘Evolutionary Biology’ (n = 153, 4.81%) and ‘Biochemistry Molecular Biology’ (n = 142, 4.46%). Among these studies, only original articles were included.

### 2.2 Methods

Scoping reviews have a different function even if there is a reporting standard for systematic reviews called the PRISMA (Preferred Reporting Items for Systematic reviews and Meta-Analyses) statement (Moher et al., 2009; Tricco et al., 2018). When it comes to providing precise answers, systematic reviews are helpful. Scoping reviews should have distinct important reporting items from systematic reviews due to the differences in aims and, therefore, in the methodological approach.

By establishing methodological guidelines that increase the value of the scientifically published literature reviews and ensure their strong reproducibility, the PICOS (Population, Intervention, Comparisons, Outcomes, and Setting) model ensures scientific diligence and objectivity of reviews (Saaiq and Ashraf, 2017). The population or themes as genomic selection in global animal breeding was emphasized using the PICOS model. As a result, by providing guidance for the mental process as it moves from the conceptual to the logical and physical stages, the PICOS model expedited search.

Bibliometrics looks at different parts of the literature on a given subject by utilizing a variety of analytical and computational techniques; it also looks at authors, institutions, countries/regions, and evaluated journals; it locates research hotspots; and it projects future research trends. Examples of these techniques include knowledge domain mapping, co-authorship analysis, and co-occurrence analysis (Garg and Garg, 2023).

Bibliometrics is an interdisciplinary field that combines mathematics, statistics, informatics, and bibliography. It is useful for doing quantitative statistical analysis on body of existing literature. A thorough reference of the history and current trends in the field’s development can be obtained by scholars through quantitative analysis of the literature utilizing scientific statistical methodologies. When bibliometric approaches are applied to a large body of reference material, they can yield a more comprehensive overview of a knowledge field than standard literature reviews (Zhang et al., 2023). According to Donthu et al. (2021), bibliometric analysis methodologies fall into two categories: science mapping and performance analysis.

The thematic analysis discusses how different topics have evolved by utilizing the writers’ keywords and their relationships. Two distinguishing features (density and centrality) set these themes apart. Density is represented by the vertical axis and centrality by the horizontal axis. Centrality quantifies the degree of linkage between various themes, while density gauges the cohesion between nodes (Esfahani et al., 2019).

An author’s place in a cooperative network can be ascertained through co-authorship analysis. In scientific and technology partnerships, the co-authorship analysis offers an insight into patterns of collaboration between individuals and organizations. It is frequently employed to comprehend and assess scientific collaboration patterns (Aziminezhad and Taherkhani, 2023).

## 3 Results

**FIGURE 1:**
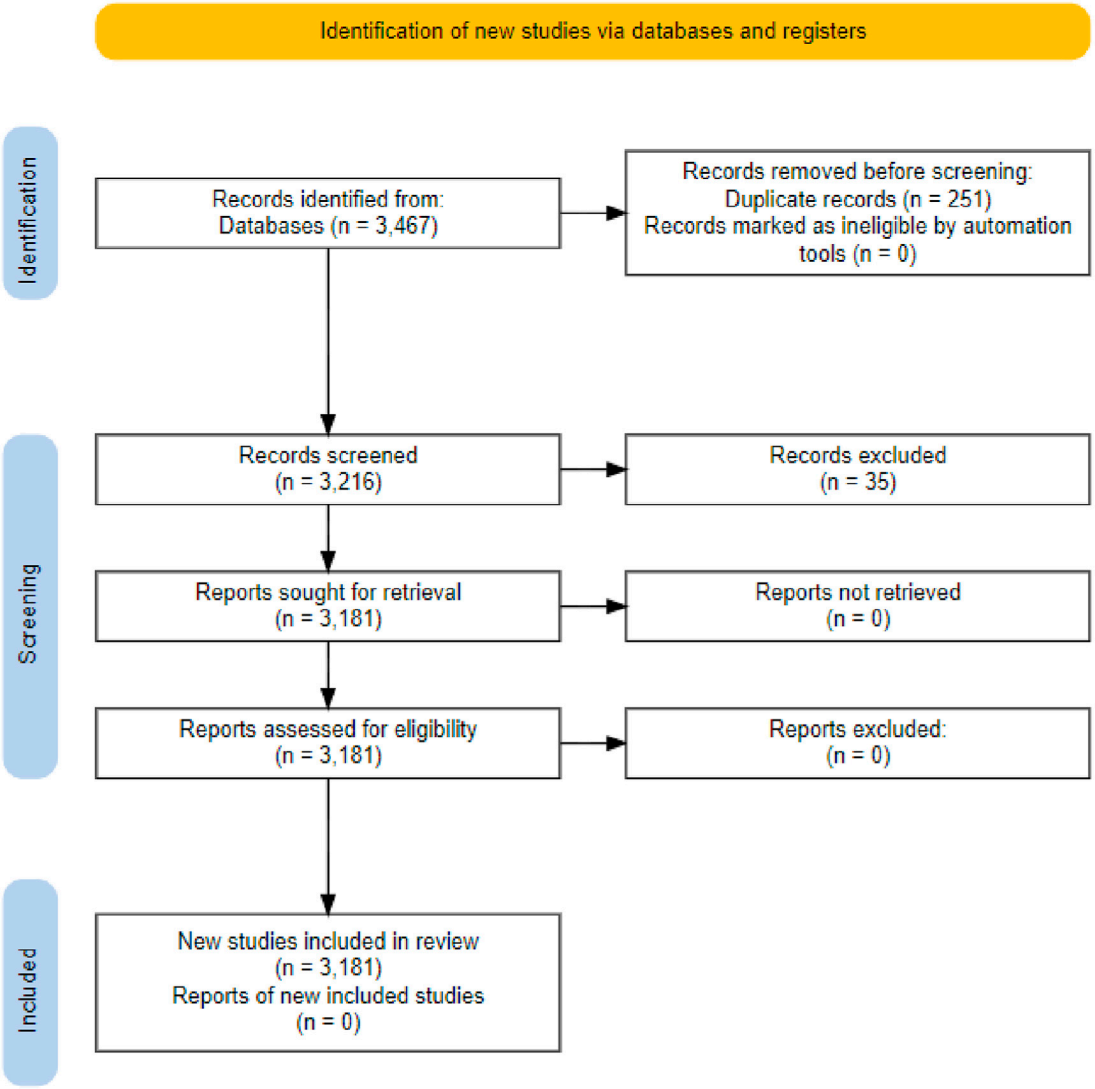
PRISMA flow diagram.

Figure 1 present a flow diagram of the studies from SCI-Esp. using the systematic procedure according to PRISMA method (Moher et al., 2009; Liberati et al., 2009; Urrútia and Bonfill, 2010). The population, interventions, comparators, outcomes, and study designs (PICOS) (Methley et al., 2014) has been used to construct eligibility criteria, as shown in Table 1.

**TABLE 1:** Eligibility criteria (PICOS).

PICOS	Description
Population	Animal breeding, genomic selection, traits, association, dairy-cattle, cattle. Population will review articles from throughout the globe
Interventions	The benefits of genomic selection in animal breeding for animal husbandry. Pedigree, growth, gene, performance
Comparator	Only at the data level of the articles: Nationality of authorship, organizational affiliation of authorship, keywords, publication year, most relevant sources, keywords co-occurrence network, trend topics
Outcomes	The bidirectional relationship between animal breeding and genomic selection, growth, prediction, performance and breeding value are emphasized
Study designs	All study types will be included: Manuscript, review, book chapter and proceedings papers

The overview of Web of Science data on genomic selection in animal breeding studies is displayed in Table 2. Total 3,181 documents released in 212 different sources between 1993 and 2024 (29 April 2024) make up the genomic selection in animal breeding databases. 1993 saw the publication of the first paper in the field of study. Article papers (n = 3,129), book chapter (n = 5), data paper (n = 5), early access (n = 12), and proceedings paper (n = 30) make up the dataset. The accumulated papers have a 16.05% annual growth rate. In addition, there are 73,447 references, 4,805 keywords and 4,746 author-keywords in the 3,181 papers on genomic selection in animal breeding. 40 articles with a single author and 10,119 authors who appeared in multi-authored papers are included in the retrieved data.

**TABLE 2:** Data Synthesis providing the essential details about the data.

Description	Results
Main information about data	
Timespan	1993:2024
Sources (Journals, Books, etc.)	212
Documents	3,181
Annual Growth Rate %	16.05
Document Average Age	5.81
Average citations per doc	28.82
References	73,447
DOCUMENT CONTENTS	
Keywords Plus (ID)	4,805
Author’s Keywords (DE)	4,746
AUTHORS	
Authors	10,119
Authors of single-authored docs	33
AUTHORS COLLABORATION	
Single-authored docs	40
Co-Authors per Doc	7.05
International co-authorships %	50.39
DOCUMENT TYPES	
Book chapter	5
Data paper	5
Early access	12
Proceedings paper	30
Journal article	3,129

Where, records identified from: Total documents idetified through WOS database search by using keywords; “genomic selection in amimal breeding”. Records removed before screening: Exclude all documents are and reviews (book chapter, conference paper, note, etc.). Records excluded: Documents that were omitted and why. Reports assessed for eligibility: Documents that were examined using bibliometrics. Records screened: Documents screened.

The PRISMA checklist was originally made up of 27 items, however it was modified to include aspects specifically for our study. A table was created from the shortened checklist’s results. The researcher separately synthesized the results derived from the PRISMA checklist, isolating the information of relevance for research purposes into a summary table (Supplementary Appendix SA1), in order to delimit and further emphasize the analysis’s findings.

**FIGURE 2:**
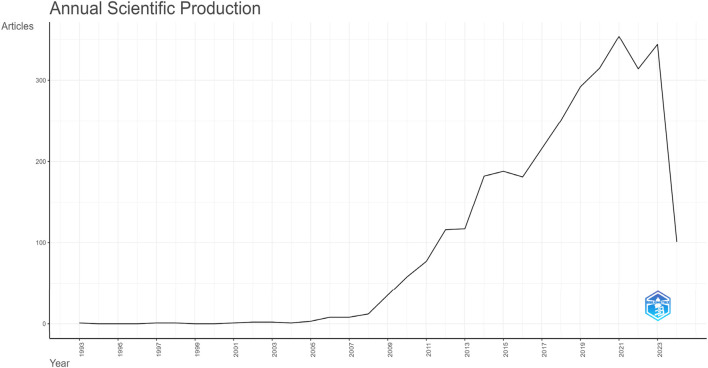
Number of Articles in the given year.

The interest in research publications on genomic selection in animal breeding has increased recently, as Figure 2 demonstrate. There were 315 published in 2020, suggesting that the field is becoming more and more popular. With 344 publications published in 2023, the publication growth is more noticeable. In 2021, there were 354 publications annually, which was a record high. In the last 10 years covering the period 2014–2024, the number of publications on the topic constituted 86.07% of all publications. As a result, the scientific contribution would advance and the academic contribution would continue to rise year.

**FIGURE 3:**
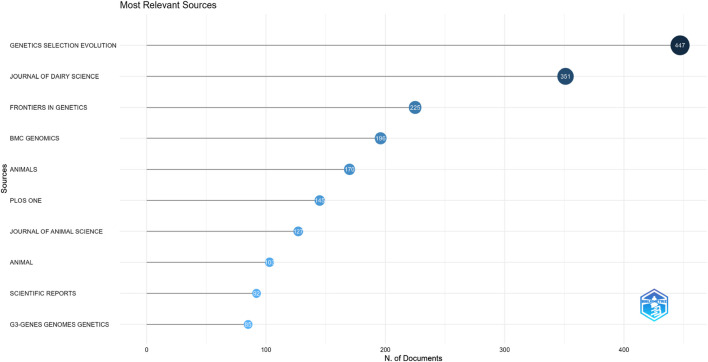
Most relevant sources.

The most important publication sources are shown in Table 3 and Figure 3. These resources would benefit the researchers who wish to study the field to keep their focus on which publications the researcher should be focused on to submit their manuscripts on genomic selection. The findings of the top 10 most pertinent sources for publications on genomic selection are shown. This conclusion has been reached based on data from Web of Science collected in April 2024. The search yielded a total of 100 journals.

**TABLE 3:** Most relevant sources.

Sources	Articles
Genetics Selection Evolution	447
Journal of Dairy Science	351
Frontiers in Genetics	225
BMC Genomics	196
Animals	170
Plos One	145
Journal of Animal Science	127
Animal	103
Scientific Reports	92
G3-Genes Genomes Genetics	85

**FIGURE 4:**
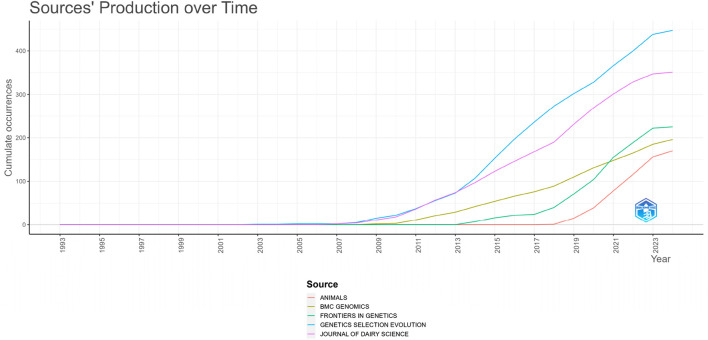
Timelines of the busiest, most productive journals.

Genetics, Selection, Evolution has 447 articles at the top of the list in Figure 3 and Table 2, followed by 351 articles from the Journal of Dairy Science. Frontiers in Genetics, with 225 publications, is not far behind. Moreover, Figure 4 shows that Journal of Dairy Science was the first publication to publish on genomic selection. The journal that is the second most active, with a growing number of articles published on the topic over time. The best papers on this subject were also published in the journals Animals, BMC Genomics, Frontiers in Genetics, Selection, and Evolution.

All of these journals are very prestigious journals that are within the scope of Science Citation Index Expanded, have a high impact factor, and generally belong to Q1 and Q2 values. Among the top 10 journals, only “G3-Genes Genomes Genetics” journal is valued at Q3, “Frontiers in Genetics” and “Scientific Reports” are valued at Q2, while the others are valued at Q1.

**FIGURE 5:**
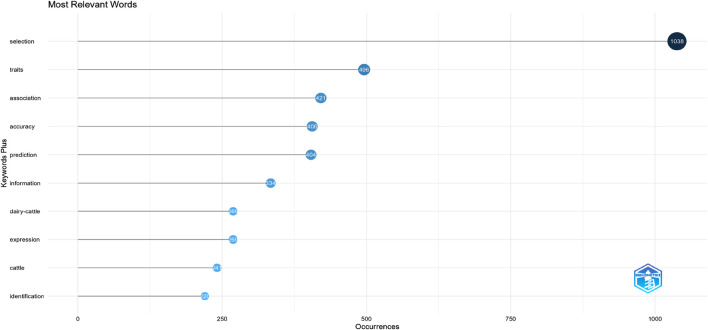
Most relevant words.

**FIGURE 6:**
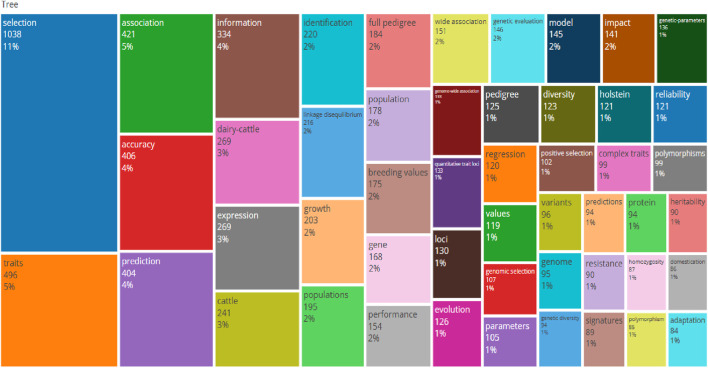
Word TreeMap of keywords correlating genomic selection.

The number of keywords by publication year is shown in Table 4 and Figures 5, 6.

**TABLE 4:** Most relevant words.

Terms	Frequency
Selection	1038
Traits	496
Association	421
Accuracy	406
Prediction	404
Information	334
Dairy-cattle	269
Expression	269
Cattle	241
Identification	220

The keywords are the high-level overview and refinement of the article’s core. High-frequency keywords in research publications may be mined and analyzed using the programme Biblioshiny. We used the keywords provided by the authors to create a frequency distribution, as shown in Figure 5. The relevant terms that appear are selection (1038), traits (496), association (421), accuracy (406), prediction (404), information (334), dairy-cattle (269), expression (269), cattle (241) and identification (220).

A Word TreeMap was created using keywords with a word frequency higher than or equal to 10 (Figure 6) using the application Biblioshiny to conduct analysis on the high-frequency keywords of the research articles. The graph shows that selection is the most often searched phrase, accounting for 11% of all the results.

**FIGURE 7:**
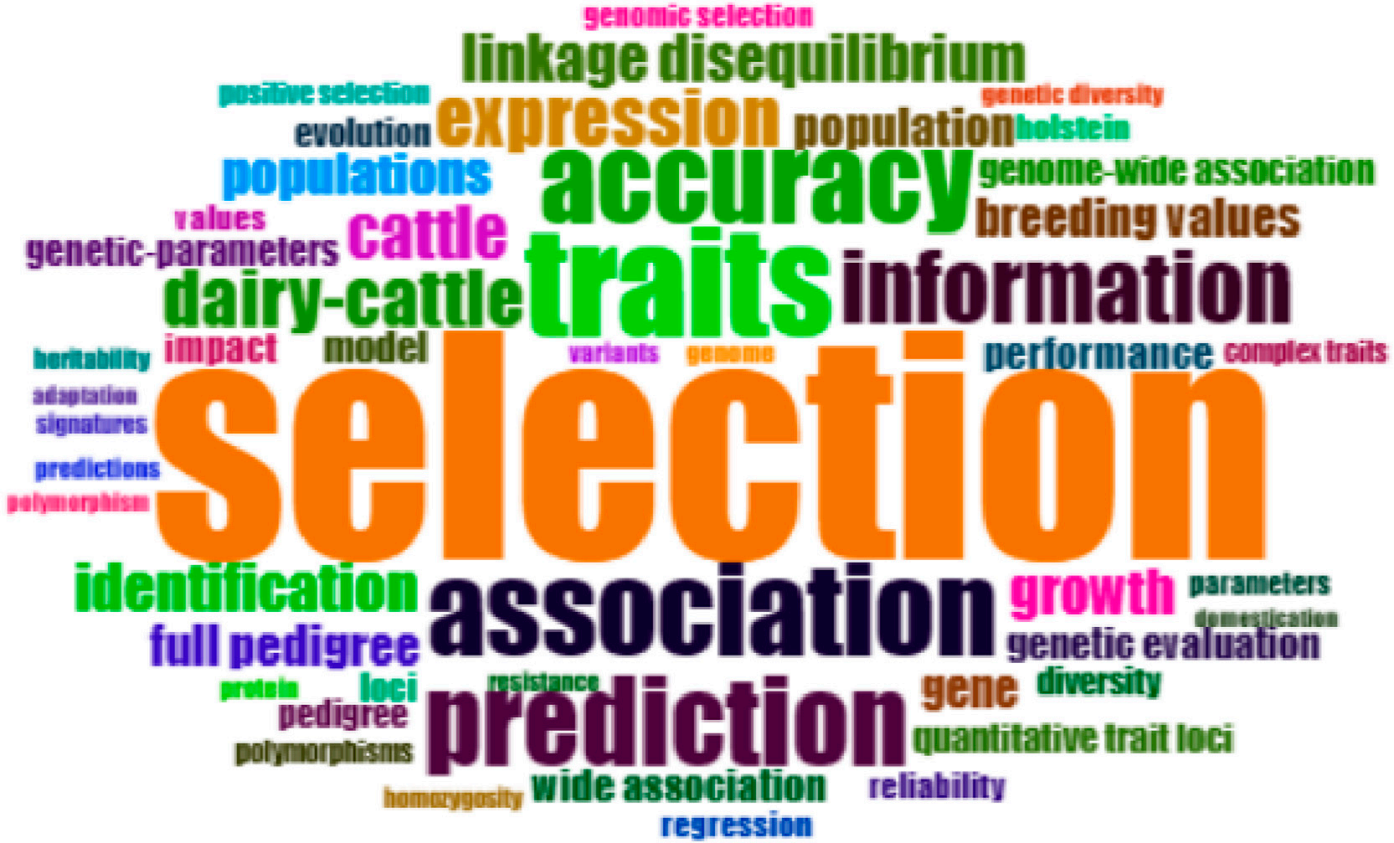
Word cloud keywords.

Examining the keywords used in the published papers is a crucial step in identifying hot themes and important academic areas. The purpose of the study is to examine these keywords as markers of problems and developments in the industry. Commonly used terms from 3,181 publications in the genomic selection of animal breeding papers are shown in the word cloud in Figure 7.

The most commonly used keywords in the 31 years’ worth of works on genomic selection are shown in Figure 7's word cloud. The findings demonstrate how breeding, genetics, and animal characteristics influence genomic selection. Selection, traits, association, accuracy, prediction, information, dairy-cattle, expression, cattle, identification, growth, populations, model, genetic evaluation, breeding values, full pedigree and performance have all attracted increasing amounts of interest between 1993 and 2024 and will likely continue to do so in the near future. The study also examined the keywords co-occurrence network patterns in genomic selection in order to gain further insight into the subject.

Among the top 10 most frequently used words, selection is used as intermediate selection of races or lines in genetic breeding strategies. Applications such as estimating the progress achieved in selection, the relationship between selection intensity and intergenerational time, the degree of accuracy in selection, estimating the progress achieved in selection are realized. The first step of genomic selection is to select a group of bulls with genotypes (single nucleotide polymorphisms, SNPs) as well as reliable estimates of their genetic value for milk production and other traits.

Regarding the expression of prediction, to date, genetic variation, genetic correlation, environment-dependent genetic interactions, chance regression, non-additive components of variance, estimation of inbreeding using SNP markers, estimation of non-additive parameters, correlations and genotype-environment interactions have been performed. In addition, estimation of breeding values using the best linear unbiased prediction (BLUP) models, the best linear unbiased prediction (BLUP) models are used to determine the sire model and animal model with the random effect in the model.

The profitability of animals is increased by breeding dairy cattle. Animal breeding work has made positive contributions to the health and welfare of animals. The relationship between genomic breeding values of young bulls and next-generation tests are carried out. Genomic breeding values (GEBV) are calculated for young bulls using genomic information.

**FIGURE 8:**
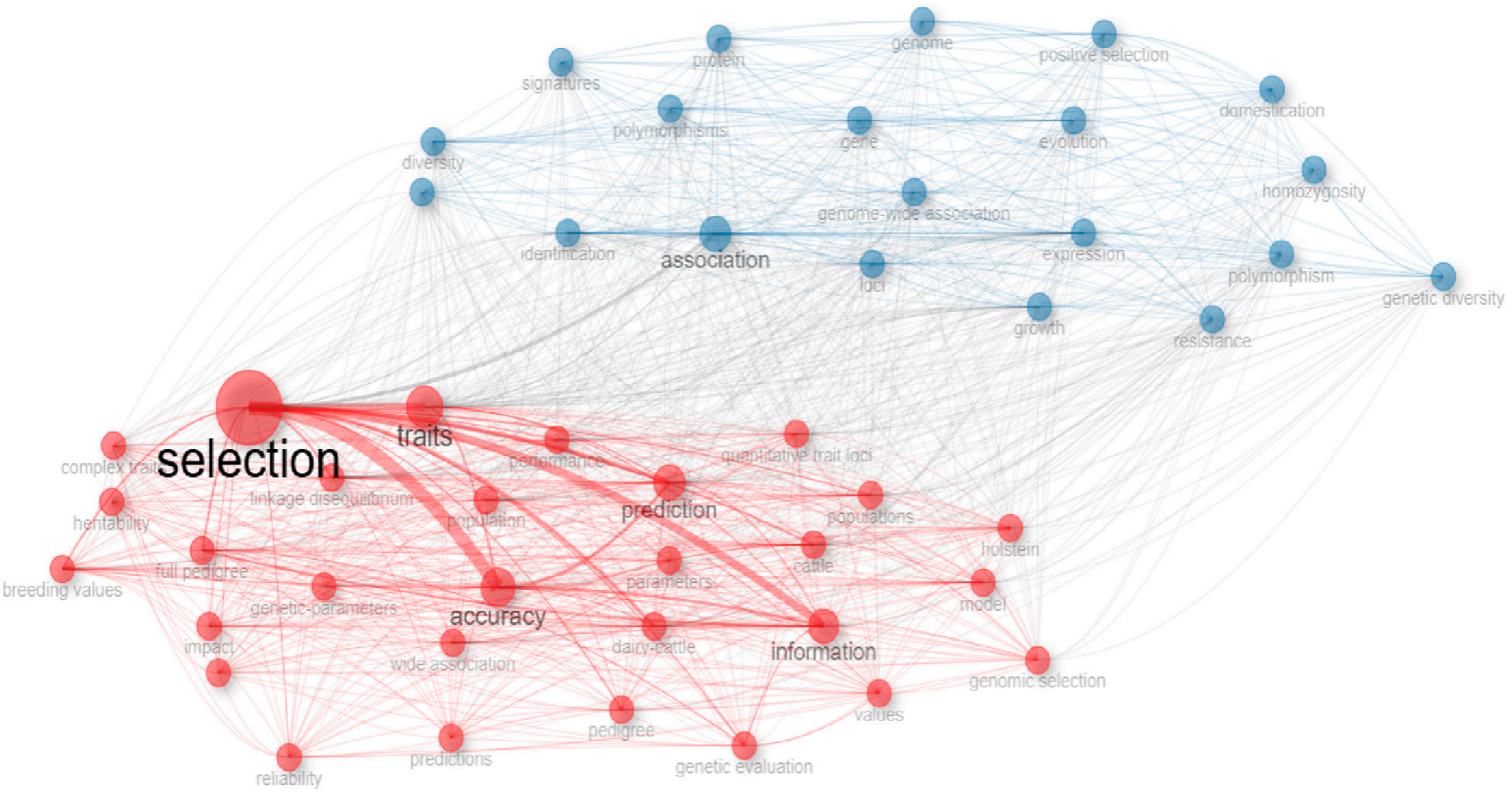
The visualization of keywords co-occurrence network.

Based on how frequently the same keywords appear in published articles, the co-occurrence of those terms is examined. Figure 8 displays the co-occurrence network of terms by year of publication. The frequency with which keywords or other terms occur together in publications is measured by co-occurrence analysis. Two separate research clusters, each with a discrete set of study subjects, are identified by this analysis.

The research fields of selection (red cluster) and association (blue cluster) are shown in Figure 8. Through a social network, collaboration networks show the shared connections between keywords. The word x word adjacency matrix, which is essentially based on the frequency of words used together, is employed in the collaboration network when collaboration networks are analyzed based on keywords. Two distinct clusters were found when the network structure was analyzed. Words were nodes in clusters, and the frequency of collaboration was indicated by the thickness of the links between words. The impact of words on the network was shown by the node’s growth.

Examining the figure’s clusters reveals that “selection” are prominent in the red cluster. The terms “traits”, “accuracy”, “prediction”, “information”, “dairy-cattle”, “cattle”, “linkage disequilibrium”, “populations”, “full pedigree”, “population”, “breeding values”, performance”, “wide association”, “genetic evaluation”, “model”, “impact”, “genetic parameters”, “quantitative trait loci”, “pedigree”, “Holstein”, “reliability”, “regression”, “values”, “genomic selection”, “parameters, “complex traits”, “predictions”, and “heritability” work in tandem with the word “selection”. Reproducibility of results is enhanced by the blue cluster’s collaboration with terms like “association,” “expression,” “identification,” “growth,” “gene,” “genome-wide association,” “loci,” “evolution,” “diversity,” “positive selection,” “polymorphisms,” “variants,” “genome,” “genetic diversity,” “protein,” “resistance,” “signatures,” “homozygosity,” “domestication,” and “polymorphism”.

**FIGURE 9:**
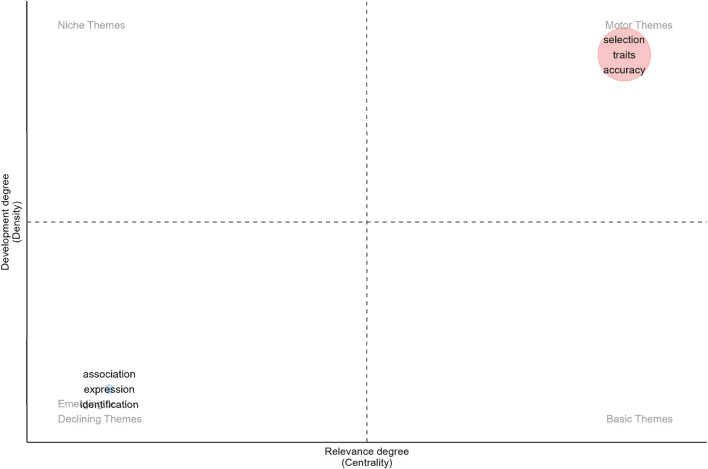
Thematic map.

The goal of the genomic selection thematic map is to facilitate comprehension of the current state of research in the field of animal breeding genomic selection and the emerging themes within it (Figure 9).

The thematic map of the genomic selection field is shown in Figure 9, with the motor themes located in the upper-right quadrant of the themes. Their great density and centrality make them stand out, suggesting that they are significant and sophisticated within the field of study. “Selection,” “traits,” and “accuracy” are the themes in this quadrant. These are well-researched ideas with structural potential.

Emerging or declining subjects are those in the lower-left quadrant. They are not developed because of their low density and centrality. Thus, there is possibility for more research on topics including “association,” “expression,” and “identification”.

**FIGURE 10:**
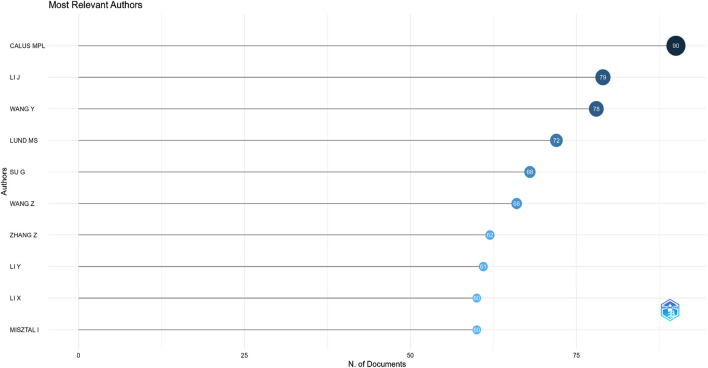
Most relevant authors.

The top 10 authors with the most articles have been identified through Web of Science. With 90 publications, Calus MPL ranked highest, followed by Li J at 79, Wang Y at 78, Lund MS at 72, Su G at 68, Wang Z at 66, Zhang Z at 62, Li Y at 61, Li X, and Misztal I at 60 (Figure 10).

**FIGURE 11:**
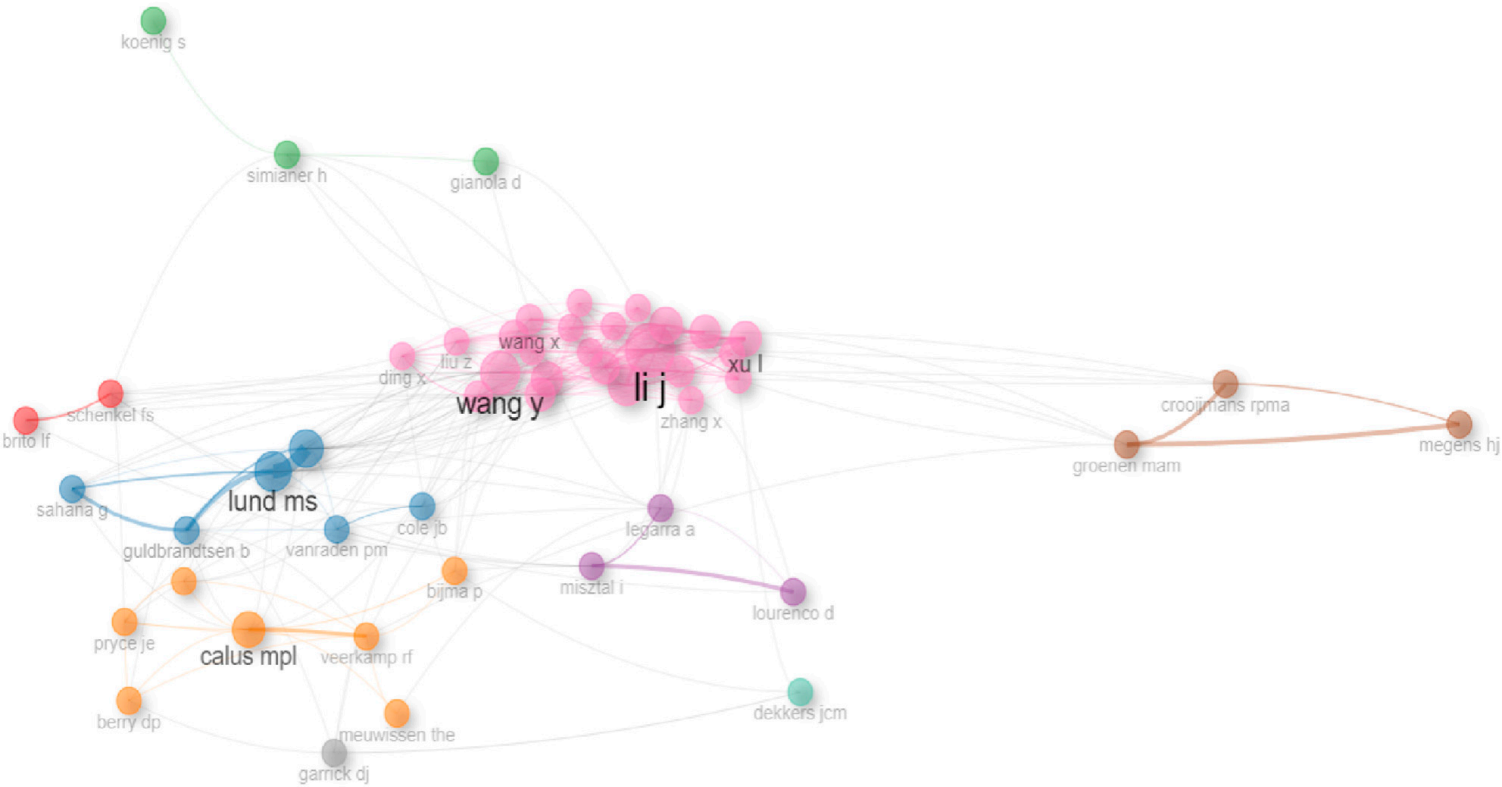
Collaboration network analysis of co-authorship.

The findings of Web of Science’s (WOS) co-authorship analysis are displayed in Figure 11. Nine colored clusters, each representing a research team, are formed from the authors.

In Figure 11, co-authorships are displayed as an overlay visualization network with colored circles labeling the elements (authors in this example) and lines connecting them. The wider the circle and label, the higher the frequency (number of articles); the colors of the circles indicate the different times at which the authors published their papers, and the distance between each element shows the correlation between them (Ni et al., 2022).

Author Co-Authorship Network In the co-authorship network, based on the 3,181 publications, a threshold of five was established as the minimum number of documents by an author, registering authors grouped into nine groups.

Cluster 1 (red) is headed by Schenkel FS and de los Brito LG. Cluster 2 (blue) is made up of Lund MS, Sahana G, Cole JB, and Guldbrandtsen B. Cluster 3 (green) is made up of Simianer H and Gianola D. Cluster 4 (pink) is made up of Wang Y, Li J, Wang X, Xu L, Ding X, and Liu Z. Cluster 5 (orange) is made up of Calus MPL, Veerkamp RF, Meuwissen THE, Berry DP, Pryce JE, and Bijma P. Cluster 6 (purple) is made up of Misztal I, Legarra A, and Leurenco D. Cluster 7 (brown) is made up of Groeman MAM, Megens HJ, and Crooijmans RPMA. Cluster 8 (green) is made up of Dekkers JCM. Cluster 9 (grey) is made up of Dekkers JCM (Figure 11).

Based on 3,181 top-published publications published between 1993 and 2024, Figure 12 shows the productivity of the top 5 institutional affiliations in genomic selection over time. The United Kingdom’s University of Edinburgh released the first edition in 1993. The papers was then produced by Iowa State University, which is located in the United States, in 1997. However, up until 2024, Aarhus University in Denmark was the most productive institution. 2018 saw them take the lead. However, the productivity increased quickly, and this growth persisted until 2024, according to the China Agricultural University and Institute of Animal Science (Stuttgart, Germany), which started writing on the topic in 2010.

**FIGURE 12:**
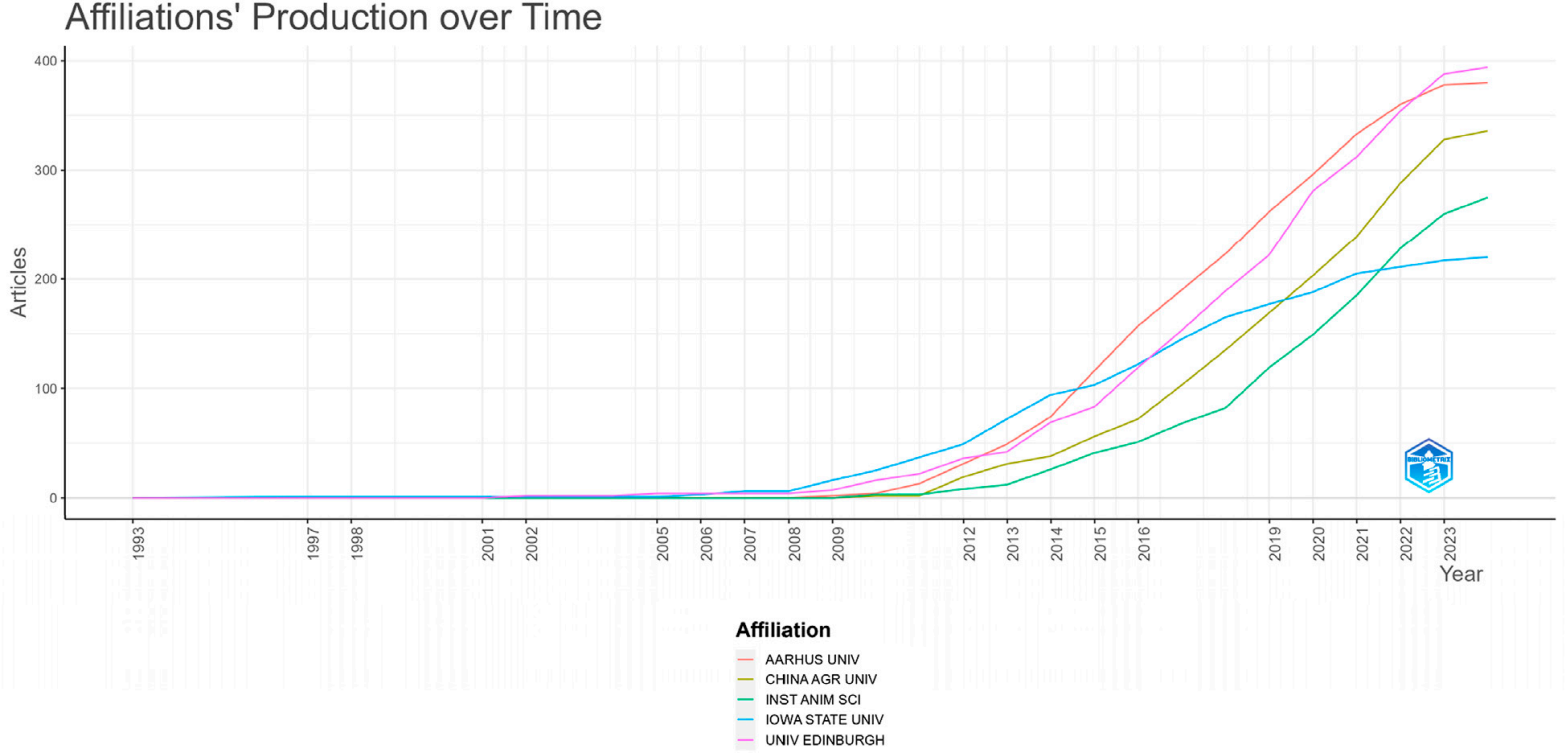
Timeline plots of the most productive institutions.

**FIGURE 13:**
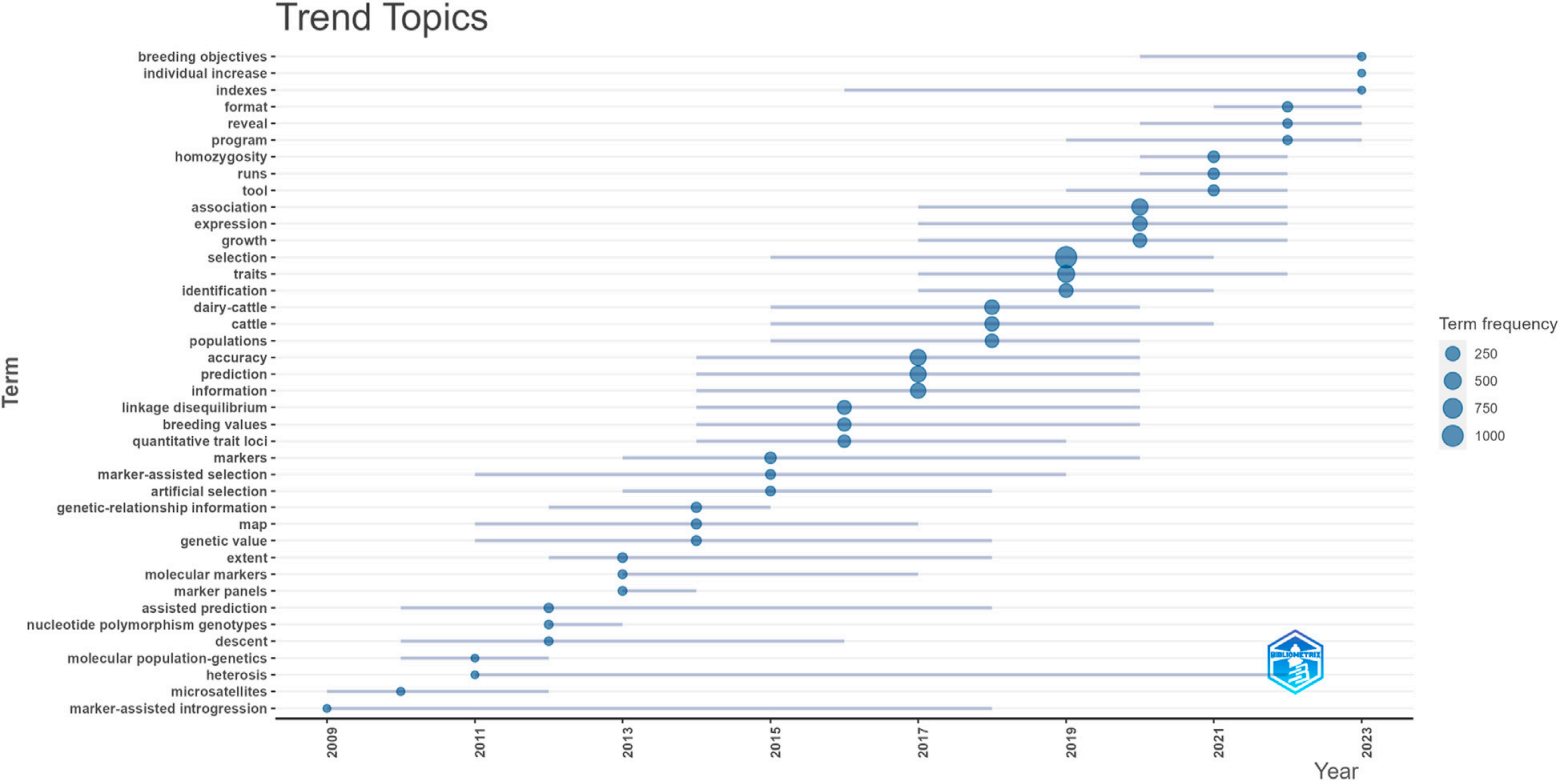
Evolution of author keyword trend topic.

The trending topic keywords that surfaced in scientific research for each year between 2009 and 2023 are shown in Figure 13.

The analysis’s findings show that the term “selection” was most frequently used in scientific research done between 2015 and 2021. As may be seen in Figure 13, the inquiry got underway in 2009. First, it identified problems with marker-assisted introgression study; then, in 2010, it concentrated on the phrases microsatellites research; in 2011, it found a lot of papers about heterosis and molecular population-genetics; and in 2012, it looked at assisted prediction, descent and nucleotide polymorphism genotypes. A studies titled “extent”, “molecular markers” and “marker panels” were conducted in 2013. By 2014, “genetic-relationship information” and “map” had become a major focus of study, with a gradual trend toward “genetic value”. In 2015, the studies of “markers”, “marker-assisted selection” and “artificial selection” were widely utilized. A lot of research were done on “linkage disequilibrium”, “breeding values” and “quantitative trait loci” in 2016. By 2017, “accuracy”, “prediction” and “information” had become the main research pillars. Studies on “accuracy, prediction and information” have given way to “dairy-cattle”, “cattle” and “populations” by 2018. By 2019, there was a great deal of interest in the relationship between “selection”, “traits” and “identification”. In the recent years, topics such as association, expression and growth (2017–2022), tool (2019–2022), homozygosity and runs (2020–2022), program (2019–2023), reveal and breeding objectives (2020–2023), format (2021–2023), indexes (2016–2023) and individual increase (2023) have taken center stage, reflecting the growing importance of animal breeding and genetic selection.

**FIGURE 14:**
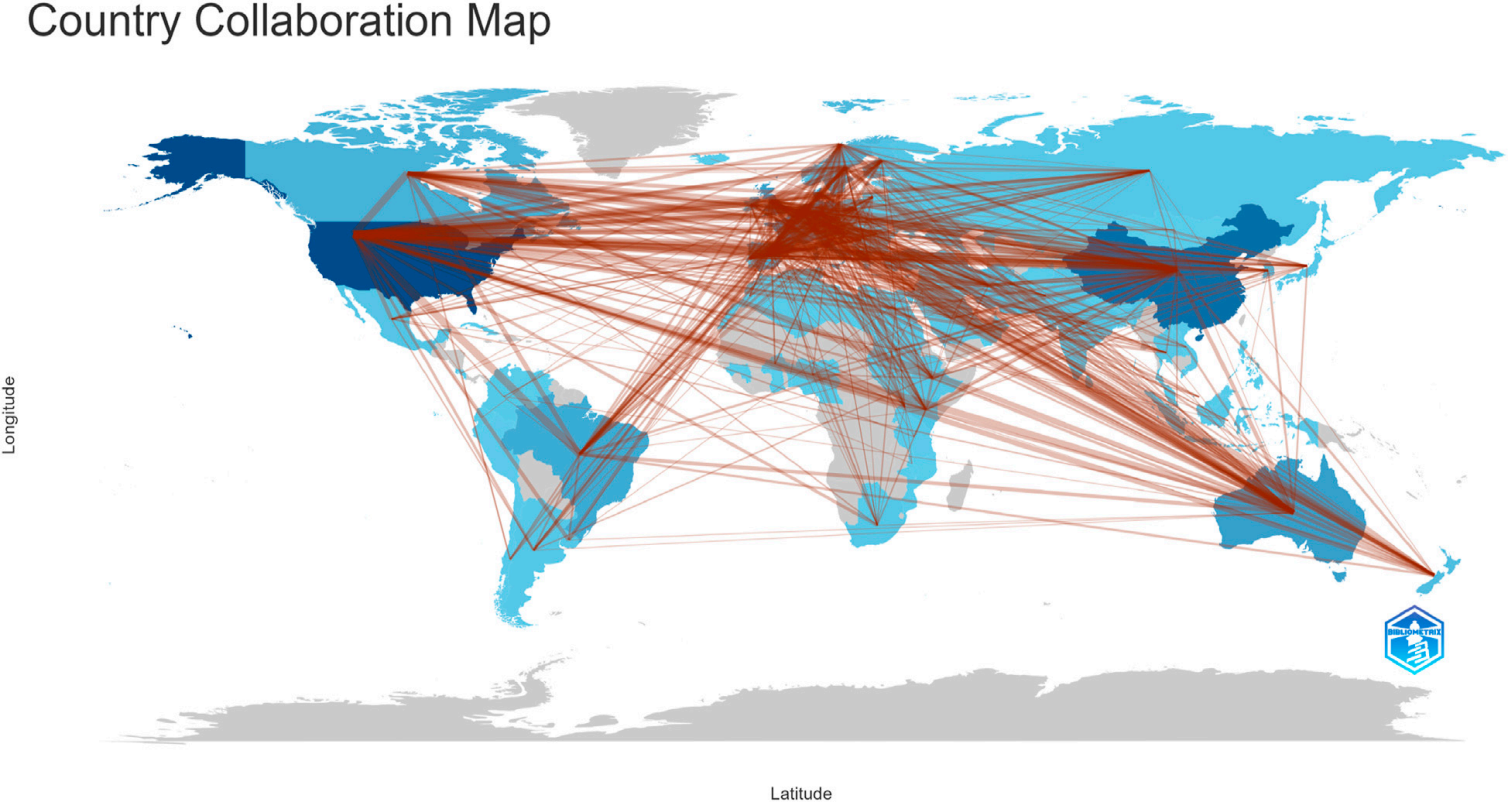
Collaboration world map.

The country/region networks show the linkages between countries/regions as well as the concentration of paper production. Utilizing the Bibliometrix program (4.0.1), Figure 14 depicts the nation/region-based networks involved in the genomic selection process in animal breeding. The degree of publishing activity in a given region is shown by the intensity of the blue hue on the nation map; a darker shade of blue indicates a larger number of published papers. On the globe, the red connecting lines represent collaborations between nations; larger lines denote more frequent collaboration. The Bibliometrix program (version 4.0.1) is used in Table 5 to provide the country/region partnerships in descending order of frequency of collaboration. China and the USA demonstrated the greatest level of cooperation with 119 relationships, followed by the USA and Brazil (101 partnerships), the USA and Canada (90 partnerships), the USA and the United Kingdom (85 partnerships), and the USA and France (78 partnerships).

**TABLE 5:** The cooperation among countries.

From	To	Frequency
China	USA	119
USA	Brazil	101
USA	Canada	90
USA	United Kingdom	85
USA	France	78
USA	Australia	73
China	Denmark	71
USA	Germany	65
USA	Netherlands	60
USA	Denmark	57

**FIGURE 15:**
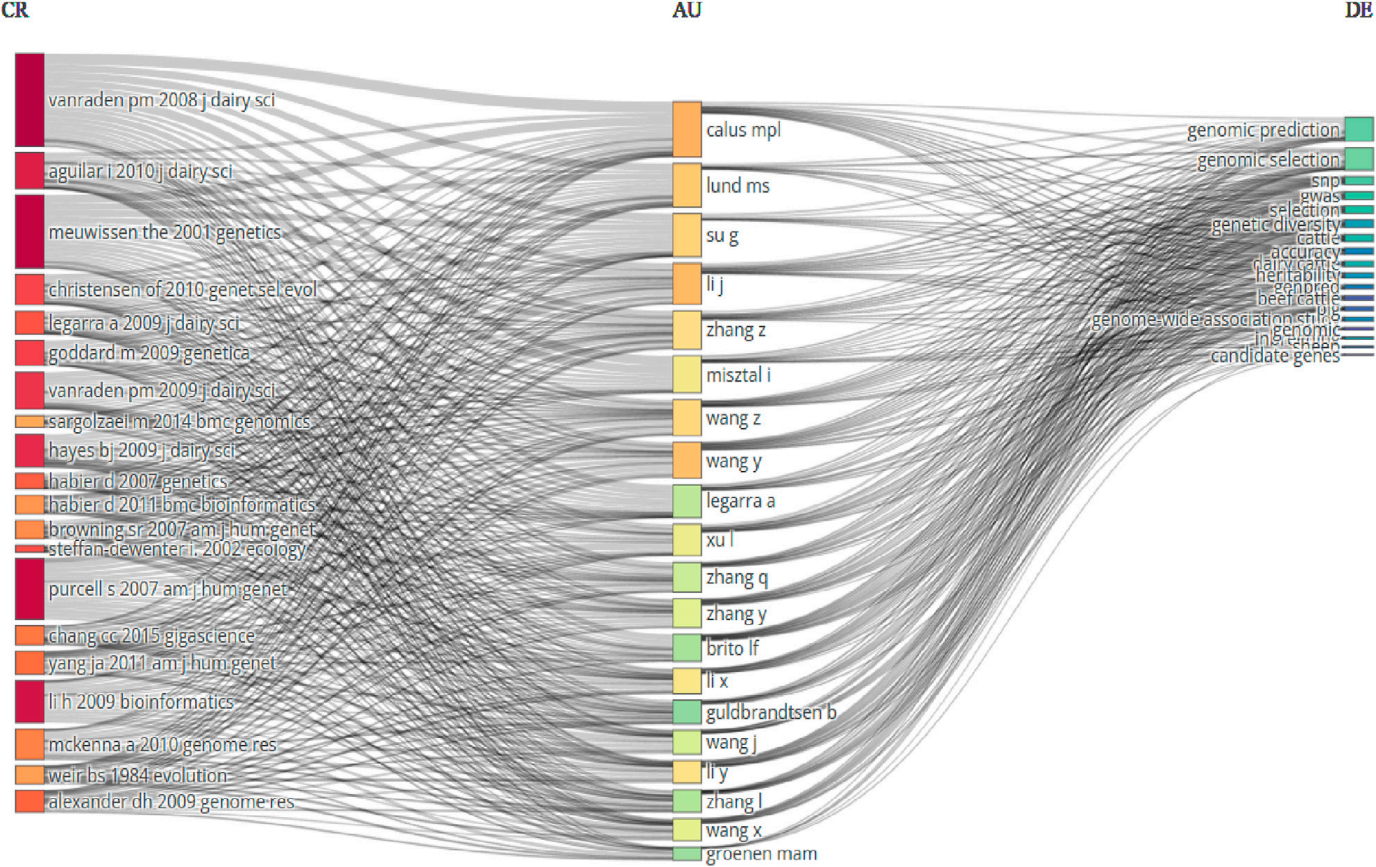
The thematic evolution of the relationship among journals (left), authors (middle), and keywords (right).

Figure 15 lists the cooperative research on the use of genomic selection in animal breeding.

Based on the data, 10,119 authors have written in this subject. Of these, only thirty-three published a paper with just one author. As a result, it shows how common collaborative research is in this area. The 7.05 co-authors per article suggest that the majority of research studies were carried out by sizable research teams. Furthermore, 50.39% of the publications generated include an international co-author, indicating a high level of engagement in the study from many nations. Figure 14 depicts a three-factor analysis of the association between authors, key terms, and journals. It demonstrates that the top five authors (Calus MPL, Lund MS, Su G, Li J, and Zhang Z) published genomic selection in animal breeding literature primarily using five key words (genomic prediction, genomic selection, SNP, GWAS, and selection). These associations and keywords are linked to five journals (Journal of Dairy Science, Genetics, Genetics Selection Evolution, Genetica and BMC Genomics).

Highly cited articles indicate that they play an important role in the collaboration with other articles. Table 6 shows the ten publications with the greatest local citations, together with their authors, sources, digital object identifier (DOI) information, publication year, and local citations. These articles were primarily published between 2007 and 2012, which could be because articles released in the last decade require more time and experiments to be confirmed. Most of these ten articles received more than 100 citations. Among these key studies, the most cited one focuses on efficient approaches for computing genomic predictions (VanRaden, 2008). According to local citation, the second-most influential publication focuses on genomic prediction in cases when some animals are not genotyped. The study’s linear mixed model specifies that a breeding value is the sum of a genomic and a polygenic genetic random effect, where genomic genetic random effects are correlated with a marker-based genomic relationship matrix and polygenic genetic random effects are correlated with the standard relationship matrix. The model’s parameters are estimated using average information REML, whereas the estimated breeding values are best linear unbiased predictions (BLUPs) (Christensen and Lund, 2010). A association matrix containing the complete pedigree and genetic data, as well as local citations, indicated the third-most influential work. The covariances among all ungenotyped individuals receive genetic information in this matrix. Unlike in the case of the naive technique, the matrix is (semi)positive definite by construction. It is discussed with numerical examples and with other phrases. With data techniques that multiply a vector by a matrix, like preconditioned conjugated gradients, Matrix H is appropriate for iteration (Legarra et al., 2009).

**TABLE 6:** Top ten papers in the field of genomic selection in animal breeding with the highest local citation score.

Document	DOI	Year	Local citations
VANRADEN PM, 2008, J DAIRY SCI	10.3168/jds.2007-0980	2008	982
CHRISTENSEN OF, 2010, GENET SEL EVOL	10.1186/1297-9686-42-2	2010	272
LEGARRA A, 2009, J DAIRY SCI	10.3168/jds.2009-2061	2009	258
HABIER D, 2007, GENETICS	10.1534/genetics.107.081190	2007	255
HABIER D, 2011, BMC BIOINFORMATICS	10.1186/1471-2105-12-186	2011	212
DAETWYLER HD, 2010, GENETICS	10.1534/genetics.110.116855	2010	192
MATUKUMALLI LK, 2009, PLOS ONE	10.1371/journal.pone.0005350	2009	190
ERBE M, 2012, J DAIRY SCI	10.3168/jds.2011-5,019	2012	172
DE ROOS APW, 2008, GENETICS	10.1534/genetics.107.084301	2008	151
GARRICK DJ, 2009, GENET SEL EVOL	10.1186/1297-9686-41-55	2009	150

The top ten authors with the highest local citation were Vanraden PM (1584), Fernando RL (1114), Misztal I (1052), Legarra A (1026), Hayes BJ (1017), Calus MPL (979), Lund MS (962), Garrick DJ (895), Habier D (764), and Christensen OF (754) (Table 7).

**TABLE 7:** List of top 10 citations authors.

Author	Local citations
VANRADEN PM	1584
FERNANDO RL	1114
MISZTAL I	1052
LEGARRA A	1026
HAYES BJ	1017
CALUS MPL	979
LUND MS	962
GARRICK DJ	895
HABIER D	764
CHRISTENSEN OF	754

The United States (26,190), China (10,231), Australia (6,107), Germany (6,071), Netherlands (5,809), Denmark (4,978), France (4,930), United Kingdom (3,437), Brazil (2,465), and Sweden (2,351) were the top ten nations with the highest total citation (Table 8).

**TABLE 8:** List of top 10 total citations countries.

Country	Total citations	Average article citations
USA	26,190	51.90
CHINA	10,231	18.60
AUSTRALIA	6,107	40.70
GERMANY	6,071	30.80
NETHERLANDS	5,809	31.90
DENMARK	4,978	33.00
FRANCE	4,930	37.60
UNITED KINGDOM	3,437	32.40
BRAZIL	2,465	18.40
SWEDEN	2,351	38.50

## 4 Discussion

In a bibliometric study on “Animal Science”, the most frequently used keywords are “heritability”, “imputation” and “linkage disequilibrium” respectively. The findings indicated that while Germany leads in producing articles for a single country, Denmark and Canada create more publications for numerous countries (Önder and Tırınk, 2022). The authors differed from the results of this study because they were doing general work on animal science. In another study, “Genetics Selection Evolution”, “Animal Genetics” and “Journal of Dairy Science” were the journals in which the studies on “Genome-Wide Association Studies” were published the most, respectively. The countries that collaborated the most in terms of publications were China, USA, Australia, Italy and Brazil, respectively (Tırınk, 2022). The findings obtained by the author and the information collected in this study were partially similar.

In a study by Yardibi et al. (2023), the most prolific and active authors were Misztal I, Calus MPL, Veerkamp RF, Lund MS, Meuwissen THE, Berry DP, Schenkel FS, Legarra A, Pryce JE and Hayes BJ. According to studies on breeding values, the United States was the most prolific nation. Germany, the US, France, Italy, and Australia had the highest centrality among the nations that constituted the global hubs of national cooperation. According to a research by Frias-De-Diego et al. (2021), South Korea, China, and the United States had the greatest rates of scientific productivity for whole genome sequences.

Research conducted prior to 2010 included gene correction, mutagenesis, crispr, gene targeting, homing endonuclease, and other related techniques, according to a study by Yu et al. (2022). After 2011, crispr, gene targeting, genome engineering, and genome editing became the subjects of studies. The primary areas of research from 2015 to 2020 included crispr/cas, gene editing, apoptosis, gene therapy, and other related topics.

The United States of America held the highest number of papers pertaining to deep learning applications of genomics methods, and it has maintained tight relationships with China and Germany. Seven categories were identified from the reference clusters of SCI articles: recombination, deep learning, variation prioritization, random forests, scRNA-seq (single-cell RNA-seq), genomic regulation, and logic regression (Zhang and Fan, 2022).

According to a study by Kaplan and Altay (2023), the publications that researchers in next-generation sequence subject prefer to use include Frontiers in Genetics, BMC Genomics, and Animals. The high influence of articles published in next-generation sequence topic was indicated by the number of citations per article. The terms “identification,” “diversity,” and “expression” was shown to be the three most often utilized keywords in next-generation sequence studies related to cattle.

In the “bibliometric analysis of research on the main genes involved in meat tenderness” study, the United States and Brazil, along with their respective institutions, lead the field both individually and in related studies based on the quantity of publications and/or citations. But the second issue was that there isn't much cooperation with the other nations. The quality of the papers is guaranteed by the fact that the Journal of Animal Science was the most popular journal and, along with the others, it had a high impact based on the various metrics assessed. Due to their impact on beef tenderness, the most influential papers, along with the trend and evolution of the primary keywords, indicate that study was concentrated on the calpain and calpastatin genes as well as single nucleotide polymorphisms (Gonzales-Malca et al., 2022). In the study by authors, ‘Journal of Animal Science’, ‘Animals’ and ‘Plos One’ journals were listed in the top 10 in the were like with this study. Among the most frequently mentioned keywords in the articles, only the word ‘cattle’ is like with this study. Large-scale single nucleotide polymorphism genomic selection studies were becoming more prevalent in an attempt to provide a more rapid and accurate explanation for the genetic variance in meat tenderness.

In an animal science research, four journals concentrated more than 60% of the scientific production: Journal of Animal Science, Journal of Dairy Science, Poultry Science and Indian Journal of Animal Sciences. Ten major theme areas were identified in the study, including animal feeding, small ruminants, animal reproduction, dairy production, meat quality, swine production, animal welfare, poultry, genetics and animal breeding, and growth factors and fatty acids. Poultry, dairy products, and animal feeding were the topic sectors with the most consistent increase. The theme areas for meat products, swine production, animal welfare, and growth factors and fatty acids are at the other end of the spectrum. Some of the subjects covered in these thematic areas such as swine production and small-ruminant production had previously been covered in other categories. It seems that before the small-ruminant theme area began to establish its own unique content, it was intimately related to eating and reproduction (Rodriguez-Ledesma et al., 2015). It differs with the results of this study.

Important topics, which is one of the interesting results of this study, have accumulated in motor and emerging and declining themes. In the bibliometric study of Banchi et al. (2022), on the contrary, most clinical topics were evaluated as niche and basic themes. Brazil, the US, Italy, Poland, and Korea ranked first and second, respectively, in terms of the quantity of documents and citations. Therefore, it differed from the results obtained in this study.

The top three core journals in a different Bibliometric Analysis study were Animals, Computers and Electronics in Agriculture, and Journal of Dairy Science. Italy, the Netherlands, and the United States were in the lead in terms of publications. The report also emphasized the growing interest in systems for producing cattle, highlighting the significance of behavioral studies in the development of precision livestock farming tools. Future themes that suggested an emphasis on environmental issues were “emissions” and “mitigation.” The most often used keywords were “machine learning,” “behavior,” “mastitis,” “milk yield,” “automatic milking system,” “dairy cow,” “animal welfare,” and “precision livestock farming” (Marino et al., 2023). The difference with the results of this study.

The difference between the findings in the studies and the findings in this study is due to the general and specific nature of the study and the scope of the research in different time intervals.

## 5 Conclusion

The bibliometric investigation of the bibliographic data was carried out on a sample of 3,181 papers about genomic selection in animal breeding published between 1993 and April 2024 using the widely accepted database in the scientific community: Web of Science.

The literature on genomic selection in animal breeding is made up mostly of studies in the fields of animals, breeding, genomics, phenotype, and models genetic, with the journals “Genetics, Selection, Evolution: GSE”, “Frontiers in Genetics”, and “Journal of Animal Breeding and Genetics” having the greatest influence in this field.

From 2010 onwards, the annual output of scientific research increased. In the most prolific nations (China and the USA), where writers work heavily on this subject, it was founded a significant concentration of papers. According to the study results, Crossa J and Gianola D, with 12 documents, and De Los Campos G, with 10, were identified as the most active writers in the field of genomic selection in animal breeding research.

This article establishes the knowledge base for future research. Despite its thorough investigation and multiple significant contributions to the body of knowledge, the study has a shortcoming. This study used only documents from the Web of Science Core Collection of Indexed Articles. Future studies should mix data from several databases. In the field of genome selection breeding in animal breeding, sustainable studies for the identification and development of ‘selection’, ‘traits’, ‘association’, ‘accuracy’ and ‘prediction’ can be prioritised.

## Data Availability

The raw data supporting the conclusions of this article will be made available by the author, without undue reservation.
